# Interest of Coffee Melanoidins as Sustainable Healthier Food Ingredients

**DOI:** 10.3389/fnut.2021.730343

**Published:** 2021-10-12

**Authors:** Amaia Iriondo-DeHond, Alejandra Rodríguez Casas, Maria Dolores del Castillo

**Affiliations:** Instituto de Investigación en Ciencias de la Alimentación (CIAL) (CSIC-UAM), Calle Nicolás Cabrera, Madrid, Spain

**Keywords:** biological properties, coffee cascara, coffee brew, coffee by-products, melanoidins, silverskin, spent coffee grounds

## Abstract

Coffee melanoidins are generated by the Maillard reaction during the thermal processes occurring in the journey of coffee from the plant to the cup (during drying and roasting). Melanoidins, the brown pigments formed as the end products of this reaction, have been reported in cascara, silverskin, spent coffee grounds, and coffee brew. The latter is one of the main natural sources of melanoidins of the daily diet worldwide. However, their presence in coffee by-products has been recently described. These complex macromolecules possess multiple health-promoting properties, such as antioxidant, anti-inflammatory, dietary fiber effect, and prebiotic capacity, which make them very interesting from a nutritional point of view. In addition, they have a great impact on the sensory profile of foods and their acceptance by the consumers. The present study is a descriptive, narrative, mini-review about the nature, structure, digestibility, properties (sensory, nutritional, and health-promoting), safety and regulatory status of melanoidins from the coffee brew and its by-products with a special emphasis on the latter.

## Introduction

**Figure 1:**
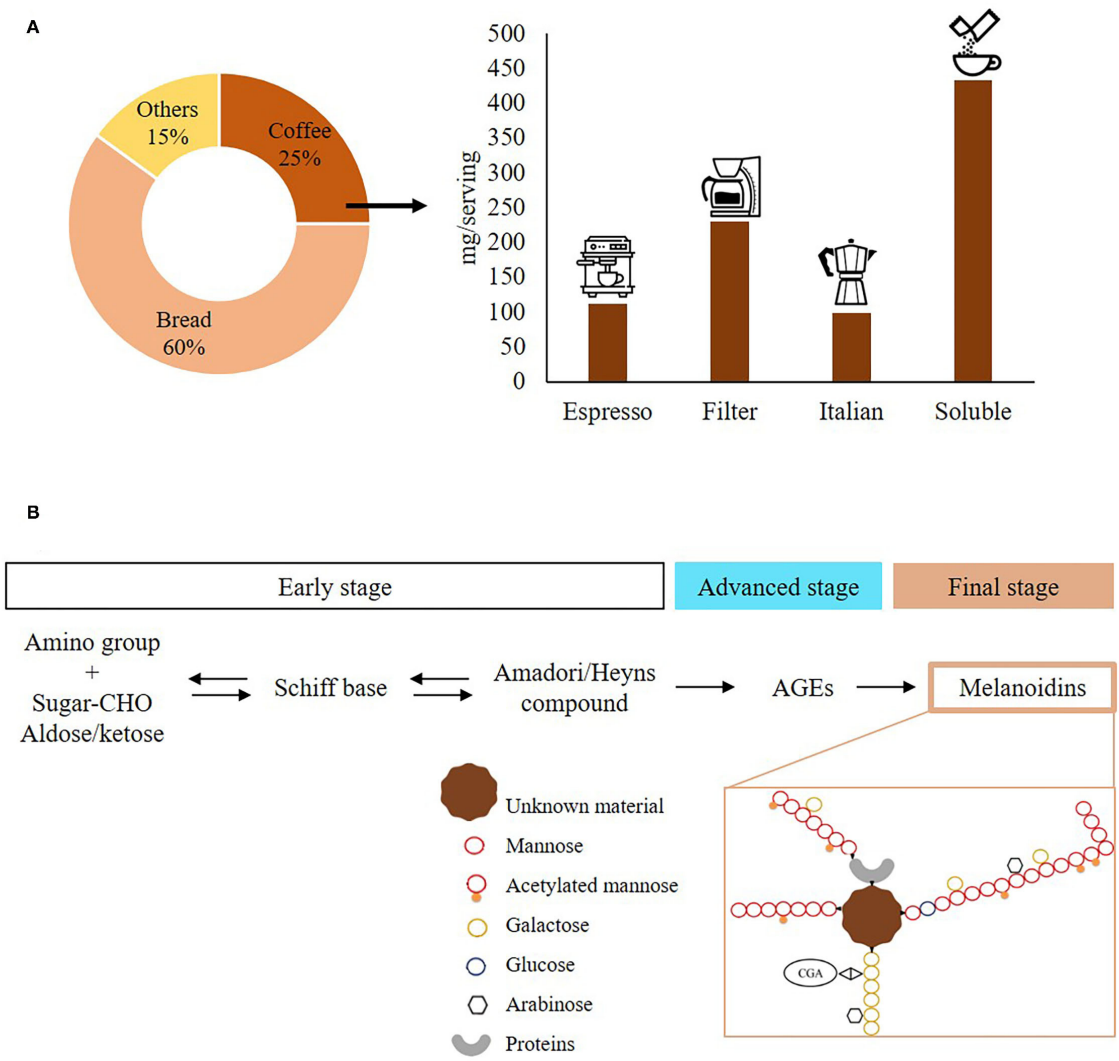
Coffee melanoidins. **(A)** Dietary intake of coffee brew melanoidins. **(B)** Pathway of formation and structure of coffee brew melanodins. Information adapted from references (1) and (2), respectively.

Coffee is one of the most extensively consumed products worldwide, and together with bakery products, it is the most important source of melanoidins in our diet (Figure 1A) (3). The estimated content of melanoidins per serving size for different preparations of coffee brew ranges from 99 to 433 mg. Therefore, an average coffee consumer (4 cups/day) can obtain 1.5 g of melanoidins from this source (1). To obtain the coffee brew, the coffee cherry undergoes different processes leading to the generation of different by-products (4). First, the coffee cherry is depulped and the by-product “cascara” is generated. Then, mucilage and parchment are produced after fermenting and milling green coffee beans, respectively. Green coffee beans are then roasted leading to the generation of the silverskin as another by-product. Finally, spent coffee grounds (SCGs) are generated after the brewing process (5). All these by-products are varied in properties and composition (Table 1) and include many interesting bioactive compounds, such as melanoidins.

**Table 1:** Step of generation during coffee processing, quantity, composition (5, 6), and biological properties of coffee by-products containing melanoidins.

**By-product**	**Cascara (wet method)**	**Silverskin**	**Spent coffee grounds**
	** 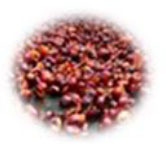 **	** 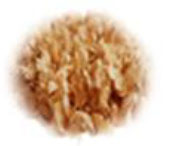 **	** 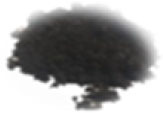 **
**Processing step**	**Pulping**	**Roasting**	**Brewing**
% By-product generated from raw material^*^/bean^**^	39–45^*^	2.08^**^	65^**^
**Composition (g/100 g)**			
Protein	10–12	16–19	13–17
Fat	2.5	2.2–3.8	1.6–2.3
Carbohydrate	44–50	62–65	71–75
Fiber	18–21	68–80	60.5
Hemicellulose	2.3	16.7	36.7
Cellulose	17.7	23.8	8.6
Lignin	17.5	28.6–30.2	24
Moisture	12.6	2.6–10	11.69
Ash	8	5–7	1.3–1.5
Caffeine	1.3	0.8–1	0.2–0.8
Chlorogenic acid	10.7	0.6–3	0.2–0.8
Melanoidins	15	17–23	13–25
**Biological properties ascribed to the melanoidins of coffee by-products**			
Biological properties	- Antioxidant (7)	- Antioxidant (8) - Dietary fiber effect (9) - Prebiotic (8)	- Antioxidant (8) - SCFAs production (10) - Prebiotic (8) - Anti-cancer (11)
Assay	- ABTS, FRAP	- ABTS, DPPH, ORAC, FRAP and HOSC - *In vivo* X-ray study - Static batch culture fermentation	- ABTS, FRAP - *In vitro* colonic fermentation - Static batch culture fermentation - SW480 cells

*ABTS: 2,2′-Azino-bis(3-ethylbenzothiazoline-6-sulfonic acid) diammonium salt; DPPH: 2,2-diphenyl-1-picryl-hydrazyl-hydrate; ORAC: Oxygen Radical Absorbance Capacity; FRAP: Ferric Reducing Antioxidant Power; HOSC: Hydroxyl Radical Scavenging Capacity*.

* *Raw material*.

** *Bean*.

Melanoidins are high–molecular weight (MW) compounds formed as a consequence of the Maillard reaction (MR) that takes place in thermally processed foods (12, 13). This reaction is the most important event that leads to the formation of melanoidins (Figure 1B), responsible for the characteristic flavor, aroma, and color of this beverage (1, 12, 14). The presence of melanoidins has been reported also in coffee by-products, such as cascara (7), silverskin (8, 9, 15), and SCGs (8, 15), generated in coffee-processing steps involving heat treatment with a different degree of intensity.

From a nutritional point of view, coffee melanoidins are considered a source of dietary fiber (16, 17). In addition, different health-promoting properties have been described for these molecules (12). Coffee brew melanoidins have been demonstrated to be more powerful antioxidants compared to melanoidins from other thermally processed foods (17). They are able to act as anticarcinogenic (18), antimicrobial, and anti-inflammatory agents (12), have antihypertensive effects by modulating the renin-angiotensin-aldosterone system (19), and can be used as a nitrogen and carbon source for the intestinal probiotic bacteria, such as bifidobacteria, exhibiting prebiotic capacity (8).

To characterize and study the health-promoting properties of melanoidins, they need to be previously isolated from the raw material. To achieve this goal, different techniques, such as dialysis, diafiltration, ultrafiltration, gel filtration chromatography, anionic exchange chromatography, and affinity chromatography, could be employed (20). Polysaccharide-type melanoidins are present in coffee and are mainly extracted by ultrafiltration, employing a 10 kDa molecular cut membrane (1, 12). This extraction process is already used in the food industry and could be applied for the extraction of these molecules from coffee (21). Although there are no studies showing that melanoidins have negative effects on human health, they can be non-covalently bound to other compounds formed during the MR that may compromise the food safety of this fraction, such as acrylamide or bioactive compounds masking their functions. To eliminate them, the melanoidin fraction can be treated with a NaCl 2 M solution overnight or be subjected to a diafiltration process (12).

The objective of the present study was to make an updated literature mini-review to highlight the potential of coffee and coffee by-product melanoidins as food ingredients. To achieve this goal, this mini-review focused on the nature, structure, digestibility, properties (sensory, nutritional, and health promoting), and safety and regulatory status of coffee and coffee by-products melanoidins.

## Materials and Methods

The present study as a narrative mini-review was conducted by a literature search consulting the Google Scholar and Web of Science databases, and the Universidad Rey Juan Carlos search engine, Brain that gives access to all the bibliographic resources of the University and the databases attached. Legislation data were consulted on the European Food Safety Authority (EFSA) webpage. Search terms related to coffee and its composition (“coffee,” “coffee by-products,” “melanoidins”) were combined with different search terms such as “health,” “novel foods,” “ingredient,” and “digestibility.” In total, and taking into account the exclusion criteria, 56 documents were selected including papers, doctoral thesis, and regulations, among others.

## Generation of Melanoidins During Coffee Processing: Nature and Structure

Coffee processing includes steps with the right conditions for the development of the MR and as a consequence, the generation of melanoidins. These melanoidins have been described in the roasted coffee bean, in the beverage, and also in the green bean, suggesting that the primary structure could be already built in the green coffee and linked with proteins, polyphenols, and MR products generated during postharvest (6, 16). But these compounds have also been reported in the by-products that originated in the different steps that lead to obtaining the coffee brew (cascara, silverskin, and SCGs) (7–9). The composition, origin, and quantity of the by-products generated along the coffee cherry processing are summarized in Table 1.

Melanoidins present in the coffee brew are extracted during the brewing process (17, 22). They have a polysaccharide core (arabinogalactans and glucomanans) and are also formed by proteins and phenolic compounds, mainly hydroxycinnamic acids (Figure 1B) (16). These melanoidins are also known as “Maillardized dietary fiber,” which is defined as “polyphenols and neoformed colored structures joined to the dietary fiber structure” during the roasting process (16). Due to the nature of this structure, melanoidins are non-digestible and considered antioxidant dietary fibers (12, 16).

Coffee cascara obtained by the wet and semi-dry methods after the depulping process is dehydrated in the sun to reduce its moisture to 10% (7). During this drying process, cascara that is composed of amino acids, proteins, and carbohydrates is subjected to moderate temperature for a long time; therefore, the necessary conditions are established for the MR to occur and as a product, melanoidins are generated (Table 1) (7). The presence of melanoidins in an aqueous extract of coffee cascara (15%) has been recently discovered (7); however, the structure of the melanoidin has not been described yet. In the same way, cascara from the dry method could be expected to have melanoidins, but there are no studies that confirm it.

Silverskin has from 17 to 21% of melanoidins (Table 1) (2). During roasting, green coffee beans are subjected to a drastic temperature increase until they reach 2% of moisture; and due to the high temperature during roasting and the composition of the coffee bean, melanoidins are generated both in the roasted coffee bean and in the silverskin (9). Melanoidins present in coffee silverskin become more complex structures as the roasting process unfolds. Low MW compounds, such as chlorogenic acids, bind non-covalently to the initial core of the melanoidin, constituted by carbohydrates, dietary fiber, polyphenols, and proteins (1, 8). Tores de la Cruz et al. (9) produced an enriched fraction of coffee silverskin melanoidins by ultrafiltration (>10 kDa). This high MW fraction obtained from coffee silverskin was composed of 75% dietary fiber and 15% melanoidins (9).

Regarding the last by-product of coffee processing, SCGs are generated after the brewing process of roasted ground coffee beans that takes place in the instant coffee industries or at the home of the individual consumer (23). SCGs have from 13 to 25% of melanoidins (Table 1) (12). The amount of melanoidins in the SCGs mainly depends on the roasting process applied to the green coffee bean and also on the extraction procedure applied to the coffee bean (1). The presence of melanoidins increases the more roasted is the bean. Nevertheless, if the roasting conditions are very intense, they start to degrade. These formation and degradation processes manifest the link between the MR and melanoidins with the time and temperature of heating (1, 20, 24). In the case of the brewing process, according to the study carried out by Fogliano and Morales (1) comparing different extraction methods, the higher is the ratio of water/coffee, the more melanoidins are extracted and thus, less remain in the SCGs (1).

Curiously, melanoidins have also been found in a coffee flower extract. More precisely, 30.2% of the dry extract obtained from the coffee flower was a high MW fraction composed of melanoidins. However, the origin of these melanoidins was found to be the process used to extract the bioactive components of the flower (25).

## Digestibility of Coffee and Coffee By-Product Melanoidins

The high MW products of the MR generally have limited bioavailability and usually result in poor absorption (26). *In vitro* and *in vivo* studies found the presence of melanoidins in rat urine, suggesting that melanoidins with less MW are partially absorbed (27). However, most melanoidins escape the superior digestion, arrive intact to the colon, and are recovered in feces (10, 17). Melanoidins can reach the colon intact and become substrates for the gut microbiota, releasing antioxidant-active molecules linked to them (13, 28–30).

During the digestion process of coffee brew melanoidins, polyphenolic structures can be released before their metabolization by the colonic microflora giving rise to the formation of metabolites with enhanced biological properties compared to the parental molecule (29). The digestibility of coffee cascara melanoidins has not yet been evaluated. Regarding the digestibility of coffee silverskin melanoidins, Castaldo et al. (31) showed that the bioaccessibility and antioxidant capacity of its polyphenols significantly increased after the colonic stage of an *in vitro* digestion (31), similar to that reported for coffee brew melanoidins. In addition, according to *in vitro* analysis carried out by Torres de la Cruz et al. (9) melanoidins from coffee silverskin present antioxidant capacity determined by ABTS (2,2′-Azino-bis(3-ethylbenzothiazoline-6-sulfonic acid) diammonium salt) and ORAC (Oxygen Radical Absorbance Capacity) and can be also defined as “maillardized dietary fiber.” *In vivo* studies carried out in male Wistar rats for 28 days showed that these melanoidins exert a dietary fiber effect, accelerating the intestinal transit of treated animals (9). Another *in vivo* study carried out also in Wistar rats reported that dietary fiber from an aqueous extract of coffee silverskin is fermentable by the gut microbiota and may have potential effects for gastrointestinal health due to the metabolites (short-chain fatty acids, SCFAs) derived from the fermentation process (32).

Cosío-Barrón et al. (10) carried out an *in vitro* study measuring the effect of the gastrointestinal conditions on the bioaccessibility of compounds, such as the high MW melanoidins of SCGs recovered by diafiltration. Results obtained in this study showed that SCGs melanoidins displayed the highest bioaccessibility from the mouth to the small intestine phase, followed by the colonic stage. On the contrary, gastric conditions reduced their bioaccessibility up to 2.7- to 4.3-folds so these melanoidins remained in the gastrointestinal tract, a key site of its antioxidant and biological action (10). These findings agree with those obtained by Perez-Burillo et al. (28) comparing the bioaccessibility of melanoidins from different foods, such as bread, coffee, or wine, among others. To achieve this goal, the study simulated an *in vitro* digestion and fermentation by the human gut microbiota of the different melanoidin fractions. As a result, melanoidins from SCGs showed that they are not fermented in the colon as occurs with bread melanoidins. However, they release the most polyphenols along with the ones present in the coffee brew (48.7 μg/g for the SCGs and 58.43 μg/g for the ones present in the brew) (28).

## Sensory, Nutritional, and Health-Promoting Properties of Melanoidins From Coffee and Coffee By-Products

Melanoidins in the coffee brew belongs to the non-volatile fraction of coffee and are able to modulate the liberation of the volatile fraction that gives the beverage its characteristic aroma (33–35) and color (22). In addition, chlorogenic acids present in the melanoidin structure may contribute to the acidic nature of the coffee brew. Together with caffeine, trigonelline, and chlorogenic acids, melanoidins have been related to coffee brew bitterness and astringency (33).

Besides their sensory properties, coffee brew melanoidins possess different biological properties such as antioxidant (36), antimicrobial (37), anti-inflammatory (38), antihypertensive (19), anticarcinogenic (39), prebiotic (28, 40), and antiglycative (41). Melanoidins present in the beverage act as an anticariogenic agent since they inhibit the adhesion of *Streptococcus mutans*, the major causative agent of dental caries in humans, almost completely at a concentration of 6 mg/ml (18). Considering the concentration of melanoidins per cup of coffee beverage (2–4 mg/ml), it is expected that they can act as a protective agent from the adhesion of this microorganism to the tooth surface (2). Walker et al. (42) have described for the first time how melanoidins from coffee affect the daily energy intake in humans. Specifically, coffee melanoidins are able to lower the blood glucose peak and insulin response due to the chlorogenic acids linked to their structure (42). These findings enhance the potential of coffee melanoidins to be used as functional ingredients in foods.

Melanoidins have been recently described for the first time in coffee cascara by Iriondo-DeHond et al. (7). Curiously, melanoidins in coffee cascara are responsible for the red/brown color of the aqueous powdered extract developed by these authors. In addition, these melanoidins have been shown to possess antioxidant capacity *in vitro* determined by ABTS and FRAP methods (Table 1) (7). Therefore, the powdered extract developed by these authors as an instant sustainable beverage can represent a great alternative to instant coffee since it also has melanoidins but has low levels of caffeine and acrylamide.

Coffee silverskin melanoidins give this by-product and its extracts the characteristic color as proved by the UV–Vis spectra analysis carried out by Iriondo-DeHond et al. (15) on the high MW fraction from a coffee silverskin extract (15). Besides, this melanoidin fraction also provides properties such as aroma and taste (43). As reported in an *in vitro* study, during the colonic stage of gastrointestinal digestion, coffee silverskin melanoidins release low MW compounds, resulting in a high antioxidant capacity (analyzed by ABTS, DPPH, ORAC, FRAP, and HOSC) compared to a non-digested sample (31, 44). These melanoidins act as “Maillardized antioxidant dietary fiber” and have been demonstrated to accelerate the small intestine transit *in vivo* (9). In addition, a study carried out by Borrelli et al. (45) showed the capacity of coffee silverskin to modulate the gut microbiota composition on *in vitro* assays. In particular, coffee silverskin was able to induce the growth of bifidobacteria instead of clostridia and *Bacteroides* spp. and thus exert a prebiotic effect that could be attributed, partially to its composition in melanoidins (45). These results were confirmed in 2015 by Jimenez-Zamora et al. (8). In addition, experiments carried out by Iriondo-DeHond et al. (46) over a coffee silverskin extract revealed the chemoprotective action of this extract. This action is a consequence of the capacity to prevent DNA damage induced by benzo-A-piren, which was attributed to chlorogenic acids in their free form or those linked to the melanoidin core (46).

Different health-promoting properties have been attributed to SCGs. Melanoidins present in SCGs also possess antioxidant capacity (8, 47). The colonic health benefits of this by-product are partially explained by the microbial degradation of the melanoidins in the colon. This degradation results in the generation of SCFAs with health-promoting properties (10). SCGs melanoidins inhibit the action of enzymes (β-glucuronidase, urease, and tryptophanase) leading to similar colonic protection effects observed for the positive control used, inulin (10). Furthermore, melanoidins from SCGs are able to inhibit the survival of SW480 human colon cancer cells by Caspase-3 activation, decreased GSH/GSSG ratio, and increase in the hypodiploid cells (SubG0), leading to apoptotic SW480 cells. Consequently, an inhibitory and reversing effect in carcinogenesis can be attributed to these melanoidins (11).

## Regulatory Status of Coffee By-Products Containing Melanoidins

Prior to the consumption of melanoidin isolates from coffee by-products, these products need to be approved for their commercialisation. To the best of our knowledge, melanoidins are generally considered safe (40, 48, 49) and there are no studies showing the potential toxicity of these molecules.

Recently, coffee cascara also known as “coffee cherry pulp” has been approved for commercialization as a “traditional food of third country” since it has been consumed as infusions for more than 25 years in Yemen, Ethiopia, and Bolivia. Therefore, after the application made by Nestlé S.A. based on many scientific studies, EFSA has stated that its use is safe and can now be placed in the E.U. market (50). With regard to coffee silverskin, EFSA does not indicate the regulatory status of this by-product. However, it may be considered not novel because it is part of the green coffee bean that is consumed as a dietary supplement, and part of it may remain partially adhered to the roasted coffee bean used to prepare the beverage. In the case of SCGs, they have been recently considered not novel due to their similarity in composition with the coffee beans (51).

In the United States, companies that are willing to commercialize these products can self-affirm that their product is “Generally Regarded As Safe” (GRAS) if there is a history of consumption with no adverse effects (52). For instance, VDF FutureCeuticals Inc. presented to the Food and Drug Administration (FDA) a report that self-affirmed that a coffee cherry extract was considered GRAS and they received a “no questions” letter from this regulatory institution that allowed the commercialization of this product (53).

Currently and to the best of our knowledge, there are no commercially available products based on isolated melanoidins obtained from the coffee brew or any of its by-products as food ingredients. However, coffee by-products are commercially available as flour, beverage, dietary supplement or honey, among others (5). Further studies are needed to establish the best food applications for coffee melanoidins. Studies carried out by our research group proposed the use of melanoidins from cascara (7) and silverskin (9) as novel healthier beverages. On the other hand, melanoidins from SCGs could be used as bakery ingredients (54, 55), for example, to make healthier biscuits (56).

## Conclusions

Melanoidins are MR products present in the coffee brew and coffee by-products (cascara, silverskin, and SCGs). The isolation of coffee by-products melanoidins is of interest for the food industry due to their technological and health-promoting properties. Consequently, coffee melanoidins represent a great opportunity for the formulation of new foods, such as beverages or bakery products, to contribute to the sustainability of the health of the consumers and also of the coffee sector. Nevertheless, it is necessary to carry out more studies to provide further and accurate information on their structure, functions, optimal conditions of isolation, and applications.

## Author Contributions

AR and AI-D: bibliographical research, manuscript development, and writing. AI-D and MC: critical revision and supervision of the manuscript. All authors contributed to the article and approved the submitted version.

## Funding

The project Nuevos conocimientos para la sostenibilidad del sector cafetero was funded by Consejo Superior de Investigaciones Científicas (CSIC) (201970E117), Novel coffee by-product beverages for an optimal health of the brain–gut axis (COFFEE4BGA) was funded by the Ministerio de Ciencia e Innovación (PID2019-111510RB-I00), and Generación de nuevos ingredientes y alimentos beneficiosos dirigidos a condiciones de riesgo y al bienestar global de personas con cáncer colorrectal (TERÁTROFO) was funded by CDTI (IDI-20190960).

## Conflict of Interest

The authors declare that the research was conducted in the absence of any commercial or financial relationships that could be construed as a potential conflict of interest.

## Publisher's Note

All claims expressed in this article are solely those of the authors and do not necessarily represent those of their affiliated organizations, or those of the publisher, the editors and the reviewers. Any product that may be evaluated in this article, or claim that may be made by its manufacturer, is not guaranteed or endorsed by the publisher.
